# Phylogenetic Analyses: A Toolbox Expanding towards Bayesian Methods

**DOI:** 10.1155/2008/683509

**Published:** 2008-04-30

**Authors:** Stéphane Aris-Brosou, Xuhua Xia

**Affiliations:** ^1^Department of Biology, Centre for Advanced Research in Environmental Genomics, University of Ottawa, Ontario, Canada K1N 6N5; ^2^Department of Mathematics and Statistics, University of Ottawa, Ontario, Canada K1N 6N5

## Abstract

The reconstruction of phylogenies is becoming an increasingly simple activity. This is mainly due to two reasons: the democratization of computing power and the increased availability of sophisticated yet user-friendly software. This review describes some of the latest additions to the phylogenetic toolbox, along with some of their theoretical and practical limitations. It is shown that Bayesian methods are under heavy development, as they offer the possibility to solve a number of long-standing issues and to integrate several steps of the phylogenetic analyses into a single framework. Specific topics include not only phylogenetic reconstruction, but also the comparison of phylogenies, the detection of adaptive evolution, and the estimation of divergence times between species.

## 1. INTRODUCTION

Human cultures have always been fascinated by their origins as a means to define their position in the world, and to justify their hegemony over the rest of the living world. However, scientific (testable) predictions about our origins had to wait for Darwin [1] and his intellectual descendents first to classify [2] and then to reconstruct the natural history of replicating entities, and hereby to kick-start the field of phylogenetics [3, 4]. Rooted in the comparison of morphological characters, phylogenies have for the past four decades focused on the relationships between molecular sequences (e.g., [4]), potentially helped by incorporating morphological information [5], in order to infer ancestor-to-descendent relationships between sequences, populations, or species.

Today, molecular phylogenies are routinely used to infer gene or genome duplication events [6], recombination [7], horizontal gene transfer [8], variation of selective pressures and adaptive evolution [9], divergence times between species [10], the origin of genetic code [11], elucidate the origin of epidemics [12], and host-parasite cospeciation events [13, 14]. As complementary tools for taxonomy (DNA barcoding: [15]), they have also contributed to the formulation of strategies in conservation biology [16]. In addition to untangling the ancestral relationships relating a group of taxa or of a set of molecular sequences, phylogenies have also been used for some time outside of the realm of biological sciences as for instance in linguistics [17, 18] or in forensics [19, 20].

Most of these applications are beyond the scope of plant genomics, but they all suggest that sophisticated phylogenetic methods are required to address most of today's biological questions. While parsimony-based methods are both intuitive and extremely informative, for instance to disentangle genome rearrangements [21], they also have their limitations due to their minimizing the amount of change [22]. These limitations become particularly apparent when analyzing distantly related taxa. A means to overcome, at least partly, some of these difficulties is to adopt a model-based approach, be in a maximum likelihood or in a Bayesian framework. These two frameworks are extremely similar in that they both rely on probabilistic models. Bayesian approaches offer a variety of benefits when compared to traditional maximum likelihood, such as computing speed (although this is not necessarily true, especially under complex models), sophistication of the model, and an appropriate treatment of uncertainty, in particular the one about nuisance parameters. As a result, Bayesian approaches often make it possible to address more sophisticated biological questions [23], which usually comes at the expense of longer computing times and higher memory requirements than when using simpler models.

Because it is not possible or even appropriate to discuss all the latest developments in a given field of study, this review will focus on a very limited number of key phylogenetic topics. Of notable exceptions, recent developments in phylogenetic hidden Markov models [24] or applications that map ancestral states on phylogenies [25] are not treated. We focus instead on the very first steps involved in *most* phylogenetic analysis, ranging from reconstructing a tree to estimating selective pressures or species divergence times. For each of these steps, some of the most recent theoretical developments are discussed, and pointers to relevant software are provided.

## 2. RECONSTRUCTING PHYLOGENETIC TREES

### 2.1. Sequence alignment

The first step in reconstructing a phylogenetic tree from molecular data is to obtain a multiple sequence alignment (MSA) where sequence data are arranged in a matrix that specifies which residues are homologous [26]. A large number of methods and programs exist [27] and most have been evaluated against alignment databases [28], so that it is possible to provide some general guidelines.

The easiest sequences to align are probably those of protein-coding genes: proteins diverge more slowly than DNA sequences and, as a result, proteins are easier to align. The rule-of-thumb is therefore first to translate DNA to amino acid sequences, then perform the alignment at the protein level, before back-translating to the DNA alignment in a final step. This procedure avoids inserting gaps in the final DNA alignment that are not multiple of three and that would disrupt the reading frame. Translation to amino acid sequences can be done directly when downloading sequences, for instance from the National Center for Biotechnology Information (NCBI: www.ncbi.nlm.nih.gov). A number of programs also allow users to perform this translation locally on their computers from an appropriate translation table (e.g., DAMBE [29], MEGA [30, 31]; see Table 1). The second step is to perform the alignment at the protein level. Again, a number of programs exist, but ProbCons [32] appears to be the most accurate *single* method [33]. An alternative for using one single alignment method is to use consensus or meta-methods, that is, to combine several methods [27]. Meta-methods such as M-Coffee can return better MSAs almost twice as often as ProbCons [34]. Finally, when the alignment is obtained at the protein level, back-translation to the DNA sequences can be performed either by using a program such as DAMBE, CodonAlign [35], or by using a dedicated server such as protal2dna (http://bioweb. pasteur.fr/seqanal/interfaces/protal2dna.html) or Pal2Nal (coot.embl.de/pal2nal).

The alignment of rRNA genes with the constraint of secondary structure has now been frequently used in practical research in molecular evolution and phylogenetics [56–60]. The procedure is first to obtain reliable secondary structure and then use the secondary structure to guide the sequence alignment. This has not been automated so far, although both Clustal [61, 62] and DAMBE have some functions to alleviate the difficulties.

What to do with other noncoding genes is still an open question, especially when it comes to aligning a large number (>100) of long (>20,000 residues) and divergent sequences (<25% identity). Some authors have attempted to provide rough guidelines to choose the most accurate program depending on these parameters [28]. However, accuracy figures are typically estimated over a large number of test alignments and may not reflect the accuracy that is expected for any particular alignment [28]. More crucially, most of the alignment programs were developed and benchmarked on protein data, so that their accuracy is generally unknown for noncoding sequences [28]. A very general recommendation is then to use different methods [63] and meta-methods. Those parts of the alignment that are similar across the different methods are probably reliable. The parts that differ extensively are often simply eliminated from the alignment when no external information can be used to decide which positions are homologous. Poorly aligned regions can cause serious problems as, for instance, when analyzing rRNA sequences in which conserved domain and variable domains have different nucleotide frequencies [60]. A simple test of the reliability of an alignment consists in reversing the orientation of the original sequences, and performing the alignment again; because of the symmetry of the problem, reliable MSAs are expected to be identical whichever direction is used to align the sequences [64]. These authors further show that reliability of MSAs decreases with sequence divergence, and that the chance of reconstructing different phylogenies increases with sequence divergence. More sophisticated methods also permit the direct measure of the accuracy of an alignments or the estimation of a distance between two alignments [65]. Applications of Bayesian inference strictly to pairwise [66] and multiple [67, 68] sequence alignment are still in their infancy.

Whichever method is used to obtain an MSA, a final visual inspection is required, and manual editing is often needed. To this end, a number of editors can be used such as JalView [69].

Because an MSA represents a hypothesis about sitewise homology at all the positions, obtaining an accurate MSA presents some circularity; an accurate MSA often necessitates an accurate guide tree, which in turn demands an accurate alignment. The early realization of this “chicken-egg” conundrum led to the idea that both the MSA and the phylogeny should be estimated simultaneously [70]. Although this initial algorithm was parsimony-based, it was already too complex to analyze more than a half-dozen sequences of 100 sites or more. Subsequent parsimony-based algorithms allowed the analysis of larger data sets [71] but still showed some limitations when sequence divergence increases. More recently, a Bayesian procedure was described and implemented in a program, BAli-Phy, where uncertainties with respect to the alignment, the tree, and the parameters of the substitution model are all taken into account [38] (see also [72]). Uncertain alignments are a potential problem in large-scale genomic studies [73] or in whole-genome alignments [74]. In these contexts, disregarding alignment uncertainty can lead to systematic biases when estimating gene trees or inferring adaptive evolution [73, 74]. However, these complex Bayesian models [38, 72, 73] still require some nonnegligible computing time and resource, and to date, their performance in terms of accuracy is still unclear.

### 2.2. Selection of the substitution model

Once a reliable MSA is obtained, the next step in comparing molecular sequences is to choose a metric to quantify divergence. The most intuitive measure of divergence is simply to count the proportion of differences between two aligned sequences (e.g., [75]). This simple measure is known as the p distance. However, because the size of the state space is finite (four letters for DNA, 20 for amino acids, and 61 for sense codons), multiple changes at a position in the alignment will not be observable, and the p distance will underestimate evolutionary distances even for moderately divergent sequences. This phenomenon is generally referred to as saturation. Corrections were devised early to help compensate for saturation. Some of the most famous named nucleotide substitution models are the Jukes-Cantor model or JC [76], the Kimura two-parameter model or K80 [77], the Hasegawa-Kishino-Yano model or HKY85 [78], the Tamura-Nei model or TN93 [79], and the general time-reversible model or GTR [80] (also called REV). Because substitution rates vary along sequences, two components can be added to these substitution models: a “+I” component that models invariable sites [78] and a “+Γ” component that models among-site rate variation either as a continuous [81] or as a discrete [82] mean-one Γdistribution, the latter being more computationally efficient. Amino acid models can also incorporate a “+F” component so that replacement rates are proportional to the frequencies of both the replaced and resulting residues [83].

Given the variety of substitution models, the first step of any model-based phylogenetic analysis is to select the most appropriate model [84, 85]. The rational for doing so is to balance bias and variance: a highly-parameterized model will describe or fit the data much better than a model that contains a smaller number of parameters; in turn however, each parameter of the highly-parameterized model will be estimated with lower accuracy for a given amount of data (e.g., [86]). Besides, both empirical and simulation studies show that the choice of a wrong substitution model can lead not only to less accurate phylogenetic estimation, but also to inconsistent results [87]. The objective of model selection is therefore not to select the “best-fitting” model, as this one will always be the model with the largest number of parameters, but rather to select the most appropriate model that will achieve the optimal tradeoff between bias and variance. The approach followed by all model selection procedures is therefore to penalize the likelihood of the parameter-rich model for the additional parameters. Because most of the nucleotide substitution models are nested (all can be seen as a special case of GTR +Γ+I), the standard approach to model selection is to perform hierarchical likelihood ratio tests or hLRTs [88]. Note that in all rigor, likelihood ratio tests can also be performed on nonnested models; however, the asymptotic distribution of the test statistic (twice the difference in log-likelihoods) under the null hypothesis (the two models perform equally well) is complicated [89] and quite often impractical. When models are nested, the asymptotic distribution of the test statistic under the null hypothesis is simply a *χ* ^2^ distribution whose degree of freedom is the number of additional parameters entering the more complex model (see [90] or [91] for applicability conditions). With the hLRT, then all models are compared in a pairwise manner, by traversing a choice-tree of possible nested models. A number of popular programs allow users to compare pairs of models manually (e.g., PAUP [51], PAML [49, 50]). Readily written scripts that select the most appropriate model among a list of named models also exist, such as ModelTest [92] (which requires PAUP), the *R* package APE [93], or DAMBE. Free web servers are also available; they are either directly based on ModelTest [94] or implement similar ideas (e.g., FindModel, available at hcv.lanl.gov/content/hcv-db/findmodel/findmodel.html). A similar implementation, ProtTest, exists for protein data [95].

However, performing systematic hLRTs is not the optimal strategy for model selection in phylogenetics [96]. This is because the model that is finally selected can depend on the order in which the pairwise comparisons are performed [97]. The Akaike information criterion (AIC) or its variant developed in the context of regression and time-series analysis in small data sets (AIC_*c*_, [98]) is commonly used in phylogenetics (e.g., [96]). One advantage of AIC is that it allows nonnested models to be compared, and it is easily implemented. However, in large data sets, both the hLRT and the AIC tend to favor parameter-rich models [99]. A slightly different approach was proposed to overcome this selection bias, the Bayesian information criterion (BIC: [99]), which penalizes more strongly parameter-rich models. All these model selection approaches (AIC, AIC_*c*_, and BIC) are available in ModelTest and ProtTest. Other procedures exist such as the Decision-Theoretic or DT approach [100]. Although AIC, BIC, and DT are generally based on sound principles, they can in practice select different substitution models [101]. The reason for doing so is not entirely clear, but it is likely due to the data having low-information content. One prediction is that, when these model selection procedures end up with different conclusions, all the selected models will return phylogenies that are not significantly different. It is also possible that applying these different criteria outside of the theoretical context in which they were developed might lead to unexpected behaviors [102]. For instance, AIC_*c*_was derived under Gaussian assumptions for linear fixed-effect models [98], and other bias correction terms exist under different assumptions [86].

All the above test procedures compare ratios of likelihood values penalized for an increase in the dimension of one of the models, without directly accounting for uncertainty in the estimates of model parameters. This may be problematic, in particular for small data sets. The Bayesian approach to model selection, called the Bayes factor, directly incorporates this uncertainty. It is also more intuitive as it directly assesses if the data are more probable under a given model than under a different one (e.g., [103]). An extension of this approach makes it possible to select the model not only among the set of named models (JC to GTR) but among all 203 nucleotide substitution models that are possible [104]. An alternative use or interpretation of this approach is to integrate directly over the uncertainty about the substitution model, so that the estimated phylogeny fully accounts for several kinds of uncertainty: about the substitution models, and the parameters entering each of these models. MrBayes (version 3.1.2) [43] implements this feature for amino acid models.

There is an element of circularity in model selection, just as in sequence alignment. In theory, when the hLRT is used for model selection, the topology used for all the computations should be that of the maximum likelihood tree. In practice, model selection is based on an initial topology obtained by a fast algorithm such as neighbor-joining [105, 106] (default setting in ModelTest) or by Weighbor [107] (default setting in FindModel) on JC distances without any correction for among-site rate variation. As mentioned above, it is known that the choice of a wrong model can affect the tree that is estimated, but it is not always clear how the choice of a nonoptimal topology to select the substitution model affects the tree that is finally estimated. Again, this issue with model choice disappears with Bayesian approaches that integrate over all possible time-reversible models as in [104].

### 2.3. Finding the “best” tree and assessing its support

Once the substitution model is selected, the classical approach proceeds to reconstruct the phylogeny [108]. This is probably one area where phylogenetics has seen mixed progress over the last five years, due to both the combinatorial and the computational complexities of phylogenetic reconstruction.

The combinatorial complexity relates to the extremely large number of tree topologies that are possible with a large number of sequences [109]. For instance, with five sequences, there are 105 rooted topologies, but with ten sequences, this number soars to over 34 million. An exhaustive search for the phylogeny that has the highest probability is therefore not practical even with a moderate number of sequences. Besides, while heuristics exist (e.g., stepwise addition [109]; see [4] for a review), almost none of these is guaranteed to converge on the optimum phylogenetic tree. The common practice is then to use one of these heuristics to find a good starting tree, and then modify repeatedly its topology more or less dramatically to explore its neighborhood for better trees until a stopping rule is satisfied [110]. The art here is in designing efficient tree perturbation methods that adaptively strike a balance between large topological modifications (that almost always lead to a very different tree with a poor score) and small modifications (that almost always lead to an extremely similar tree with lower score). Some of today's challenges are about choosing between methods that successfully explore large numbers of trees but that can be costly in terms of computing time [110], and methods that are faster but may miss some interesting trees [53]. Several programs such as Leaphy, PhyML, and GARLI[41] are among the best-performing software in a maximum likelihood setting. In a Bayesian framework, the basic perturbation schemes were described early [36] and recently updated [111]. Three popular programs are MrBayes, BAMBE [36], and BEAST [39]. Among all these programs and approaches, PHYML, GARLI, and BEAST are probably among the most efficient programs in terms of computational speed, handling of large data sets and thoroughness of the tree search.

A first aspect of the computational complexity relates to estimating the support of a reconstructed phylogeny. This is more complicated than estimating a confidence interval for a real-valued parameter such as a branch length, because a tree topology is a graph and not a number. The classical approach therefore relies on a nonstandard use of the bootstrap [112]. However, the interpretation of the bootstrap is contentious. Bootstrap proportions *P* can be perceived as testing the correctness of internal nodes, and failing to do so [113], or 1–*P* can be interpreted as a conservative probability of falsely supporting monophyly [114]. Since bootstrap proportions are either too liberal or too conservative depending on the exact interpretation given to these values [115], it is difficult to adjust the threshold below which monophyly can be confidently ruled out [116]. Alternatively, an intuitive geometric argument was proposed to explain the conservativeness of bootstrap probabilities [117], but the workaround was never actually used in the community or implemented in any popular software. The introduction of Bayesian approaches in the late 1990s [36, 118] suggested a novel approach to estimate phylogenetic support with posterior probabilities. Clade or bipartition posterior probabilities can be relatively fast to compute, even for large data sets analyzed under complicated substitution models [119]. As in model selection, they have a clear interpretation as they measure the probability that a clade is correct, given the data and the model. But as with bootstrap probabilities, some controversies exist. Early empirical studies found that posterior probabilities of highly supported nodes were much larger than bootstrap probabilities [120], and subsequent simulation studies supported this observation (e.g., [121–124]). Some of these differences can be attributed to an artifact of the simulation scheme that was employed [125], but more specific empirical and simulation studies show that prior specifications can dramatically impact posterior probabilities for trees and clades [115, 126, 127]. In the simplest case, the analysis of simulated star trees with four sequences fails to give the expected three unrooted topologies with equal probability (1/3, 1/3, 1/3) but returns large posterior probabilities for an arbitrary topology [115, 126], even when infinitely long sequences are used [128, 129] ([130]). This phenomenon, called the star-tree paradox [126], seems to disappear when polytomies are assigned nonzero prior probabilities and when nonuniform priors force internal branch length towards zero [129]. The second issue surrounding Bayesian phylogenetic methods is about their convergence rate. A theoretical study shows that extremely simple Markov chain Monte Carlo (MCMC) samplers, the technique used to estimate posterior probabilities, could take an extremely long time to converge [131]. In practice, however, MCMC samplers such as those implemented in MrBayes are much more sophisticated. In particular, they include different types of moves [111] and use tempering, where some of the chains of a single run are heated, to improve mixing [43]. As a result, it is unclear whether they suffer from extremely long convergence times. It is also expected that current convergence diagnostic tools such as those implemented in MrBayes would reveal convergence problems [132]. Finally, it is also argued that these controversies such as exaggerated clade support, inconsistently biased priors, and the impossibility of hypothesis testing disappear altogether when posterior probabilities at internal nodes are abandoned in favor of posterior probabilities for topologies [133] (see Section 2.4 below).

The most fundamental aspect of the computational complexity in phylogenetics is due to the structure of the phylogenies: these are trees or binary graphs on which computations are nested and interdependent, which makes these computations intractable or NP-hard [134]. As a result, it is difficult to adopt an efficient “divide and conquer” approach, where a large complicated problem would be split into small simpler tasks, and to take advantage of today's commodity computing by distributing the computation over multicore architectures or heterogeneous computer clusters. Current strategies are limited to distributing the computation of the discrete rate categories (when using a “+Γ” substitution model) and part of the search algorithm [54], or simply to distribute different maximum likelihood bootstrap replicates [53, 54] or different MCMC samplers to available processors [44].

### 2.4. Comparisons of tree topologies

Science proceeds by testing hypotheses, and it is often necessary to compare phylogenies, for instance to test whether a given data set supports the early divergence of gymnosperms with respect to Gnetales and angiosperms (the anthophyte hypothesis), or whether the Gnetales diverged first (the Gnetales hypothesis) [135, 136]. Because of the importance of comparing phylogenies, a number of tests of molecular phylogenies were developed early. The KH test was first developed to compare two random trees [137]. However, this test is invalid if one of the trees is the maximum likelihood tree [138]. In this case, the SH test should be used [139]. Because the SH test can be very conservative, an approximately unbiased version was developed: the AU test [140]. PAUP and PAML only implement the KH and SH tests; CONSEL [40] also implements the AU test. A Bayesian version of these tests also exists [141], but the computations are more demanding.

Indeed, the Bayesian approach to hypothesis testing relies on computing the probability of the data under a particular model. This quantity is usually not available as a close-form equation, and it must be approximated numerically. The most straightforward approximation is based on the harmonic mean of the likelihood sampled from the posterior distribution [142]. This approximation was described several times in the context of phylogenies [141, 143] and is available from most Bayesian programs such as MrBayes or BEAST. However, the approximation is extremely sensitive to the behavior of the MCMC sampler [52, 142]: if extremely low-likelihood values happen to be sampled from the posterior distribution, the harmonic mean will be dramatically affected. To date, a couple of more robust approximations have been described and were shown to be preferable to the harmonic mean estimator [52]. The first is based on thermodynamic integration [52] and is available in PhyloBayes (see Table 1). The second approximation [144] is based on a more direct computation [145], but its availability is currently limited to one specific model of evolution.

### 2.5. More realistic models

While model selection is fully justified on the ground of the bias-variance tradeoff, it should not be forgotten that all these models are simplified representations of the actual substitution process and are all therefore wrong. Stated differently, if AIC selects the GTR +Γ+I to analyze a data set, it should be clear that this conclusion does not imply that the data evolved under this model. All model selection procedures measure a relative model fit. One way to estimate adequacy or absolute model fit is to perform a parametric bootstrap test [146]: *first*, the selected model is compared with a multinomial model by means of a LRT whose test statistic is *s* (twice the log-likelihood difference); the following steps determine the distribution of *s* under the null hypothesis that the selected model was the generating model; *second*, the selected model is used to simulate a large number of data sets; *third*, the model selection procedure (LRT) is repeated on each simulated data set, and the corresponding test statistics s* are recorded; *fourth*, the *P*-value is estimated as the number of times, the simulated s* test statistics are more extreme (>, for a one-sided test) than the original value of *s*. The results of such tests suggest that the selected substitution model is generally not an adequate representation of the actual substitution process [85]. Of course, we do not need a model that incorporates all the minute biological features of evolutionary processes. As argued repeatedly (e.g., [147]), we need *useful* models that capture enough of reality of substitution processes to make accurate predictions and avoid systematic biases such as long-branch attraction [148].

More realistic models are obtained by accommodating heterogeneities in the evolutionary process at the level of both sites (space) and lineages (time). The simplest site-heterogeneous model is one, where the aligned data are partitioned, usually based on some prior information. For instance, first and second codon positions are known to evolve slower than third codon positions in protein-coding genes, or exposed residues might evolve faster than buried amino acids in globular proteins. A number of models were suggested to analyze such partitioned data sets (e.g., [149]); these models are implemented in most general-purpose software (e.g., PAML, PAUP, MrBayes) and can be combined with a “+Γ+I” component. A different approach consists in considering that sites can be binned in a number of rate categories; the use of a Dirichlet prior process then makes it possible both to determine the appropriate number of categories and to assign sites to these categories; the application of this method to protein-coding genes was able to recover the underlying codon structure of these genes [150]. However, several studies suggest that evolutionary patterns can be as heterogeneous within a priori partitions as among partitions [37, 151].

Lineage-heterogeneous models or heterotachous models [152] have attracted more attention. In one such approach, different models of evolution are assigned to the different branches of the tree [153], which can make these models extremely parameter-rich. Such a large number of parameters can potentially affect the accuracy of the phylogenetic inference (see the “bias-variance tradeoff” above) and present computational issues (long running times, large memory requirements, and convergence issues). Several simplifications can be made. One assumes that some sets of branches evolve under a particular process [153]. But now these branches must be assigned a priori, and both the determination of the number of sets and their placement on the tree can be difficult (but see Section 4 below for a solution to a similar question). At the other end of the spectrum of heterotachous models lies the simplest model known as the covarion model [154], where sites can either be variable along a branch, or not, and can switch between these two categories across time (e.g., [155], also described in a Bayesian framework [156]).

Between these two extremes are mixture models, which extend the covarion model by allowing more categories of sites. A number of formulations exist, where each site is assumed to have been generated by either several sets of branch lengths [157, 158] or by several rate matrices [37, 96, 151]. One particularity of these models is that they give a semiparametric perspective to the phylogenetic estimation: if a single simple model cannot approximate a complex substitution process, the hope is that mixing several simple substitution models makes our models more realistic. In some applications, mixture models can also be used to avoid underestimating uncertainty, first when choosing a single model of evolution and then ignoring this uncertainty when estimating the phylogeny. The mixing therefore involves fitting at each site several sets of branch lengths, or several substitution models to the data, and combining these models using a certain weighting scheme. The difference between the numerous mixture models that have been described lies in the choice of the weight factors, and how these are obtained. In one approach, known as model averaging, the weights are determined a priori. A first possibility is to assume that all the models are equally probable, which does not work with an infinite number of models (individual weights are zero in this case). More critically in phylogenetics, this assumption is not coherent for nested models since larger models should be more likely than each submodel. A second possibility is to weight the models with respect to their probability of being the generating model given the data. For practical purposes, this posterior probability can be approximated by Akaike weights [96]. The difficulty here is that model averaging requires analyzing the data even for models that, a posteriori, turn out to have extremely small probabilities or weights. This may be seen as a waste of resources (computing time and storage space).

### 2.6. Integrated Bayesian approaches

Mixture models can work within the framework of maximum likelihood, but the treatment of the weight factors is complicated. A sound alternative is to resort to a fully Bayesian approach. A prior distribution is set on the weight factors, and a special form of MCMC sampler whose Markov chain moves across models with different numbers of parameters, a reversible-jump MCMC sampler (RJ-MCMC), is constructed. The advantage of RJ-MCMC samplers is that they allow estimating the phylogeny while integrating over the uncertainty pertaining to the parameters of the substitution model and even integrating over the model itself [104]. Mixture models are available in BayesPhylogenies [37] for nucleotide models. Another Bayesian mixture model, named CAT for CATegories, was developed to analyze amino acid alignments. The CAT model recently proved successful in a number of empirical [159, 160] and simulation [161] studies in avoiding the artifact known as long-branch attraction [148]. This model is freely available in the PhyloBayes software (see Table 1).

All these models assume that each site evolve independently. The independence assumption greatly simplifies the computations, but is also highly unrealistic. Models that describe the evolution of doublets in RNA genes [162], triplets in codon models [163, 164], or other models with local or context dependencies [165–167] exist, but complete dependence models are still in their infancy and, so far, have only been implemented in a Bayesian framework [168, 169]. One particularly interesting feature of this approach is that complete dependence models incorporate information about the three-dimensional (3D) structure of proteins and therefore permit the explicit modeling of structural constraints or of any other site-interdependence pattern [170]. The incorporation of 3D structures also allows the establishment of a direct relationship between evolution at the DNA level and at the phenotypic level. This link between genotype and phenotype is established via a proxy that plays the role of a fitness function which, in retrospect, can be used to predict amino-acid sequences compatible with a given target structure, that is, to help in protein design [171].

## 3. DETECTING POSITIVE SELECTION

Fitness functions are however difficult to determine at the molecular level. In addition, while examples of adaptive evolution at the morphological level abound, from Darwin's finches in the Galapagos [172] to cichlid fishes in the East African lakes [173], the role of natural selection in shaping the evolution of genomes is much more controversial [147, 174]. First, the neutral theory of molecular evolution asserts that much of the variation at the DNA level is due to the random fixation of mutations with no selective advantage [175]. Second, a compelling body of evidence suggests that most of the genomic complexities have emerged by nonadaptive processes [176]. A number of statistical approaches exist either to test neutrality at the population level or to detect positive Darwinian evolution at the species level [147]. A shortcoming of neutrality tests is their dependence on a demographic model [177] and their sensitivity to processes of molecular evolution such as among-site rate variation [178]. They also do not model alternative hypotheses that would permit distinguishing negative selection from adaptive evolution. The development of demographic models based on Poisson random fields [179] and composite likelihoods [180] makes it possible both to estimate the strength of selection and to assess the impact of a variety of scenarios on allele frequency spectra [9]. But demographic singularities such as bottlenecks can still generate spurious signatures of positive selection [180, 181].

When effective population sizes are no longer a concern, for instance in studies at or above the species level, the detection of positive selection in protein-coding genes usually relies on codon models [163, 164] (see [182] for a review including methods based on amino-acid models). Codon models permit distinguishing between synonymous substitutions, which are likely to be neutral, and nonsynonymous substitutions, which are directly exposed to the action of selection. If synonymous and nonsynonymous substitutions accumulate at the same rate, then the protein-coding gene is likely to evolve neutrally. Alternatively, if nonsynonymous substitutions accumulate slower than synonymous substitutions, it must be because nonsynonymous substitutions are deleterious and this suggests the action of purifying selection. Conversely, the accumulation of nonsynonymous substitutions faster than synonymous substitutions suggests the action of positive selection. The nonsynonymous to synonymous rate ratio, denoted *ω* = *d*_*N*_/*d*_*S*_, is therefore interpreted as a measure of selection at the protein level, with *ω* = 1, <1 and >1 indicating neutral evolution, negative or positive selection, respectively. This ratio is also denoted *K*_*a*_/*K*_*s*_, in particular in studies that rely on counts of nonsynonymous and synonymous sites (e.g., [183]). An extension exists to detect selection in noncoding regions [184], and a promising phylogenetic hidden Markov or phylo-HMM model permits detection of selection in overlapping genes [185].

These rate ratios can be estimated by a number of methods implemented in MEGA, DAMBE, HyPhy [42], and PAML. The most intuitive methods, called counting methods, work in three steps: (i) count synonymous and nonsynonymous sites, (ii) count the observed differences at these sites, and (iii) apply corrections for multiple substitutions [186]. Counting methods are however not optimal in the sense that most work on pairs of sequences and therefore, just like neighbor-joining, fail to account for all the information contained in an alignment. In addition, simulations suggest that counting methods can be sensitive to a variety of biases such as unequal transition and transversion rates, or uneven base, or codon frequencies [187]. Counting methods that incorporate these biases perform generally better than those that do not, but the maximum likelihood method still appears more robust to sever biases [187]. In addition, the maximum likelihood method that accounts for all the information in a data set has good power and good accuracy to detect positive selection [188, 189].

However, the first studies using these methods found little evidence for adaptive evolution essentially because they were averaging *ω* rate ratios over both lineages and sites [147]. Branch models were then developed [190, 191] quickly followed by site models [192–196] and by branch-site models [189, 197]. All these approaches, as implemented in PAML, rely on likelihood ratio tests to detect adaptive evolution: a model where adaptive evolution is permitted is compared with a null model where *ω* cannot be greater than one. Simulations show that some of these tests are conservative [189], so that detection of adaptive evolution should be safe as long as convergence of the analyses is carefully checked [198], including in large-scale analyses [199]. If the model allowing adaptive evolution explains the data significantly better than the null model, then an empirical Bayes approach can be used to identify which sites are likely to evolve adaptively [192]. The empirical Bayes approach relies on estimates of the model parameters, which can have large sampling errors in small data sets. Because these sampling errors can cause the empirical Bayes site identification to be unreliable [200], a Bayes empirical Bayes approach was proposed and was shown to have good power and low-false positive rates [201]. Full Bayesian approaches that allow for uncertain parameter estimates were also proposed [202]. Yet, simulations showed that they did not improve further on Bayes empirical Bayes estimates [203], so that the computational overhead incurred by full Bayes methods may not be necessary in this case. One particular case, where a Bayesian approach is however required, is to tell the signature of adaptive evolution from that of recombination, as these two processes can leave similar signals in DNA sequences. Indeed, simulations show that recombination can lead to false positive rates as large as 90% when trying to detect adaptive evolution [204]. The codon model with recombination implemented in OmegaMap [48] can then be used to tease apart these two processes (e.g., see [205]).

## 4. ESTIMATING DIVERGENCE TIMES BETWEEN SPECIES

The estimation of the dates when species diverged is often perceived to be as important as estimating the phylogeny itself. This explains why so-called “dating methods” were first wished for when molecular phylogenies were first reconstructed [206]. In spite of over four decades of history, molecular dating has only recently seen new developments. One of the reasons for this slow progress is that, unlike the other parts of phylogenetic analysis, divergence times are parameters that cannot be estimated directly. Only sitewise likelihood values and distances between pairs of sequences are identifiable, that is, directly estimable. Distances are expressed as a number of substitutions per site (sub/site) and can be decomposed as the product of two quantities: a rate of evolution (sub/site/unit of time) and a time duration (unit of time). As a result, time durations and, likewise, divergence times cannot be estimated without making an additional assumption on the rates of evolution. The simplest assumption is to posit that rates are constant in time, which is known as the molecular clock hypothesis [207]. This hypothesis can be tested, for instance, with PAUP or PAML, by means of a likelihood ratio test that compares a constrained model (clock) with an unconstrained model (no clock). These two models are nested, so that twice the log-likelihood difference asymptotically follows a *χ* ^2^ distribution. If *n* sequences are analyzed, the constrained model estimates *n* − 1 divergence times, while the unconstrained model estimates 2*n* − 3 branch lengths. The degree of freedom of this test is then (2*n* − 3) − (*n* − 1) = *n* − 2 [4]. The systematic test of the molecular clock assumption on recent data shows that this hypothesis is too often untenable [208].

The most recent work has then focused on relaxing this assumption, and three different directions have emerged [209]. A first possibility is to relax the clock *globally* on the phylogeny, but to assume that the hypothesis still holds *locally* for closely related species [210–212]. Recent developments of these local clock models now allow the use of multiple calibration points and of multiple genes [213], the automatic placement of the clocks on the tree [214] and the estimation of the number of local clocks [209]. PAML can be used for most of these computations. However, local clock models still tend to underestimate rapid rate change [209]. The second possibility to relax the global clock assumption is to assume that rates of evolution evolve in an autocorrelated manner along lineages and to minimize the amount of rate change over the entire phylogeny. The most popular approach in the plant community is Sanderson's penalized likelihood [215], implemented in r8s [55]. This approach performs well on data sets for which the actual fossil dates are known [216] but still tends to underestimate the actual amount of rate change [209].

Bayesian methods appear today as the emerging approach to estimate divergence times. Taking inspiration from Sanderson's pioneering work [217], Thorne et al. developed a Bayesian framework where rates of evolution change in an autocorrelated manner across lineages [45–47]: the rate of evolution of a branch depends on the rate of evolution of its parental branch; the branches emanating from the root require a special treatment. These Bayesian models work by modeling how rates of evolution change in time (rate prior), and how the speciation/population process shapes the distribution of divergence times (speciation prior). These prior distributions can actually be interpreted as penalty functions [45, 209], and they can have simple or more complicated forms [218]. The Multidivtime program [45–47] is extremely quick to analyze data thanks to the use of a multivariate normal approximation of the likelihood surface. It assumes that rates of evolution change following a stationary lognormal prior distribution. Further work suggested that it might not always be the best performing rate prior [218–220], but these latter studies had two potential shortcomings: (i) they were based on a speciation prior that was so strong that it biased divergence times towards the age of the fossil root [219, 221], and (ii) they used a statistical procedure, the posterior Bayes factor [222], that is potentially inconsistent. One potential limitation of the Bayesian approach described so far is its dependence on one single tree topology, which must be either known ahead of time or estimated by other means. Recently, Drummond et al. found a way to relax this requirement by positing that rates of evolution are uncorrelated across lineages, while all the branches of the tree are constrained to follow exactly the same rate prior [223]. As a result, their approach is able to estimate the most probable tree (given the data and the substitution model), the divergence times and the position of the root even without any outgroup or without resorting to a nonreversible model of substitution [224]. Drummond et al. further argue that the use of explicit models of rate variation over time might contribute to improved phylogenetic inference [223]. In addition, when the focus is on estimating divergence times, a recent analysis suggests that this uncorrelated model of rate change could outperform the methods described above to accommodate rapid rate change among lineages [209]. Implemented in BEAST, this approach offers a variety of substitution models and prior distributions and presents a graphic user interface that will appeal to numerous researchers [39].

## 5. CHALLENGES AND PERSPECTIVES

With the advent of high-throughput sequencing technologies such as the whole-genome shotgun approach by pyrosequencing [225], fast, cheap, and accurate genomic information is becoming available for a growing number of species [226]. If low coverage limits the complete assembly of many genome projects, it still allows the quick access to draft genomes for a growing number of species [227]. As a result, phylogenetic inference can now incorporate large numbers of expressed sequence tags (ESTs), genes [228], and occasionally complete genomes [229]. The motivation for developing these so-called phylogenomic approaches is their presumed ability to return fully resolved and well-supported trees by decreasing both sampling errors [230] and misleading signals due for instance to horizontal gene transfer [231] or to hidden paralogy [232]. In practice, these large-scale studies can give the impression that incongruence is resolved [228], but they also can fail to address systematic errors due to the use of too simple models [233]. If the genes incorporated in phylogenomic studies are often concatenated to limit the number of parameters entering the model, it remains important to allow sitewise heterogeneities [234]. If partition models can reduce systematic biases [234], Bayesian mixture models such as CAT [151] appear to be robust to long-branch attraction [159], a rampant issue in phylogenomics [235]. All together, the accumulation of genomic data and these latest methodological developments seem to make the reconstruction of the tree of life finally within reach. In comparison, dating the tree of life is still in its infancy, even if a number of initiatives such as the TimeTree server are being developed [236]. These resources are limited to some vertebrates but will hopefully soon be extended to include other large taxonomic groups such as plants. To achieve this goal, however, phylogenetic studies should systematically incorporate divergence times, as is now routine in some research communities (e.g., [237]). This joint estimation of time and trees is today facilitated by the availability of user-friendly programs such as BEAST. The near future will probably see the development of mixture models for molecular dating and more sophisticated models that integrate most of the topics discussed here from sequence alignment to detection of sites under selection into one single but yet user-friendly [238] toolbox.

## Figures and Tables

**Table 1:** *List of programs cited in this review*. GUI: graphic user interface; ML: maximum likelihood; PL: penalized likelihood.

Name	Method	Platform	GUI	Inference	Reference
BAMBE	Bayes	DOS, MacOS, Unix	No	Tree	[36]
BayesPhylogenies	Bayes	DOS, MacOS, Unix	No	Tree	[37]
BAli-Phy	Bayes	DOS, MacOS, Unix	No	Simultaneous alignment and tree	[38]
BEAST	Bayes	Windows, MacOS, Unix	Yes	Tree, times	[39]
CONSEL	ML	DOS, MacOS, Unix	No	Tree comparison	[40]
DAMBE	Distances, parsimony, ML	Windows	Yes	Tree	[29]
GARLI	ML (Genetic Algorithm)	Windows, MacOS, Unix	Yes	Tree	[41]
HyPhy	ML	Windows, MacOS, Unix	Yes	Tree, selection, recombination, tree comparison,	[42]
MEGA	Distances, parsimony	Windows	Yes	Tree, times	[30, 31]
MrBayes	Bayes	DOS, MacOS, Unix	No	Tree, selection	[43, 44]
Multidivtime	Bayes	DOS, MacOS, Unix	No	Times	[45–47]
OmegaMap	Bayes	DOS, MacOS, Unix	No	Simultaneous selection and recombination	[48]
PAML	ML	DOS, MacOS, Unix	No	Tree, tree comparison, times, selection	[49, 50]
PAUP∗	Distances, parsimony, ML	DOS, MacOS, Unix	No	Tree	[51]
PhyloBayes	Bayes	DOS, MacOS, Unix	No	Tree, tree comparison	[52]
PHYML	ML	DOS, MacOS, Unix	No	Tree	[53]
RAxML	ML	DOS, MacOS, Unix	No	Tree	[54]
r8s	PL	DOS, MacOS, Unix	No	Times	[55]
